# Circulating CD3 ^+^ CD8 ^+^ T Lymphocytes as Indicators of Disease Status in Patients With Early Breast Cancer

**DOI:** 10.1002/cam4.70547

**Published:** 2025-01-03

**Authors:** Han‐Kun Chen, Yi‐Ling Chen, Wei‐Pang Chung, Zhu‐Jun Loh, Kuo‐Ting Lee, Hui‐Ping Hsu

**Affiliations:** ^1^ Department of Surgery Chi Mei Medical Center Tainan Taiwan; ^2^ Department of Nursing Meiho University Pingtung Taiwan; ^3^ Department of Health and Nutrition Chia Nan University of Pharmacy and Science Tainan Taiwan; ^4^ Department of Oncology, College of Medicine National Cheng Kung University Hospital, National Cheng Kung University Tainan Taiwan; ^5^ Center of Applied Nanomedicine National Cheng Kung University Tainan Taiwan; ^6^ Department of Surgery, College of Medicine National Cheng Kung University Hospital, National Cheng Kung University Tainan Taiwan; ^7^ Department of Biochemistry and Molecular Biology College of Medicine, National Cheng Kung University Tainan Taiwan

**Keywords:** biomarker, breast cancer, CCL2, circulating immune cells, cytotoxic T lymphocytes

## Abstract

**Background:**

Identifying breast cancer markers with superior sensitivity, cost‐effectiveness, and practicality is imperative. Circulating immune cells and plasma cytokines hold promise as potential breast cancer markers.

**Aims:**

To search and validate subtype of circulating immune cells and plasma cytokines as biomarkers for breast cancer patients.

**Materials & Methods:**

Using flow cytometry, we investigated circulating immune cell profiles in patients with breast cancer and healthy controls. To validate clinical observations, an orthotopic breast cancer model was established.

**Results:**

We analyzed 19 healthy controls and 27 patients (22 testing group and 5 validation group) with breast cancer and revealed distinct populations, including CD3+CD4+ T lymphocytes, cytotoxic T lymphocytes (CTLs; CD3+CD8+), polymorphonuclear myeloid‐derived suppressor cells (PMN‐MDSCs), and monocytic (M)‐myeloid‐derived suppressive cells. Patients with breast cancer exhibited reduced CD3+CD4+ T lymphocyte, CD3+CD8+ CTL, and CD33+CD15− M‐MDSC levels compared with healthy controls. Diminished CD3+CD8+ CTL levels correlated with advanced cancer grade, extensive intraductal components, and positive lymphatic tumor emboli. Treatment effects included decreased T lymphocyte/PMN‐MDSC levels, contrasting with elevated circulating CD3+CD8+ cell levels posttreatment, subsequently declining upon recurrence in the validation group. Elevated plasma chemokine (C–C motif) ligand 2 (CCL2) levels distinguished patients with breast cancer from healthy controls. Our orthotopic model supported decreased circulating CD3+CD8+ CTL levels in cancer‐bearing mice, followed by a postresection increase.

**Discussion:**

We found that circulating CD3+CD8+ CTL levels decreased in patients with breast cancer, increased after treatment, and decreased again upon recurrence.

**Conclusion:**

Circulating CD3+CD8+ CTL emerged as promising prognostic biomarkers and therapeutic targets in breast cancer management.

Circulating CD3^+^CD8^+^ cell levels were lower in breast cancer patients, elevated posttreatment, and subsequently declining upon recurrence. Elevated plasma chemokine (C–C motif) ligand 2 (CCL2) levels distinguished patients with breast cancer from healthy controls. In summary, circulating CD3^+^CD8^+^ CTL and plasma CCL2 levels emerged as promising dual‐purpose biomarkers and therapeutic targets in breast cancer management.

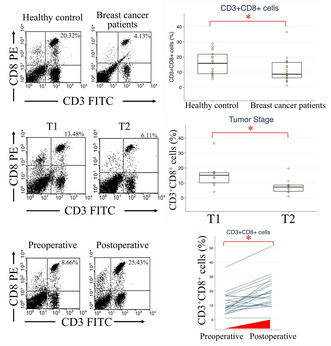

## 1 Introduction

Breast cancer, a complex and heterogeneous disease, is grouped into four subtypes [Luminal A, Luminal B, Her‐2–enriched, and triple‐negative breast cancer (TNBC)] according to immunohistochemical markers, including estrogen receptor, progesterone receptor, human epidermal growth factor receptor 2 (Her‐2/neu), and cell proliferative index Ki‐67 [1]. Standard breast cancer treatments encompass surgery, chemotherapy, radiotherapy, endocrine therapy, targeted therapy, and immunotherapy; however, despite standard therapy, recurrence can follow. Importantly, recurrence is potentially driven by cancer–immune cell interaction.

The tumor microenvironment, a complex blend of cells and molecules, is pivotal in breast cancer progression and metastasis. Components of breast cancer include tumor‐infiltrating lymphocytes (TILs), tumor‐associated macrophages, cancer‐associated fibroblasts, endothelial cells, myeloid‐derived suppressive cells (MDSCs), the extracellular matrix, secreted cytokines, and exosomes [2]. Cytokines, including tumor necrosis factor‐α, interleukin (IL)‐6, IL‐10, IL‐12, IL‐17, transforming growth factor‐β, chemokine (C–C motif) ligand 2 (CCL2; also known as monocyte chemoattractant protein 1), chemokine (C–X–C motif) ligand 1, and macrophage migration inhibitory factor, influence tumor development [3]. Additionally, immune metagene scores predict survival and recurrence, particularly regarding intrinsic subtypes and estrogen receptor status in highly proliferative tumors [4]. Breast cancer cells manipulate the immune system via cytokines and receptor expression, evading immune surveillance in a process termed immune escape. Immunosuppression regulates tumor‐infiltrating immune cells, with the process involving suppressive factors, regulatory T cells, macrophage differentiation, and natural killer cell suppression. Direct cell–cell contact also inhibits tumor‐specific cytotoxic T cells. Disseminated cancer cells, especially those in systemic circulation (circulating tumor cells (CTCs)) or bone marrow/lymph nodes, display potent immune escape, leading to metastasis and death [5].

Immune response dynamics drive breast cancer progression. Ductal carcinoma in situ lacks metastatic potential but hosts an active immune milieu, enriched in cytotoxic T cells and diverse T‐cell receptors. In contrast, invasive carcinoma displays immune suppression, fewer cytotoxic cells, higher programmed death‐ligand 1 (PD‐L1) expression levels, and less diverse T‐cell receptors [6]. High TIL levels correlate with improved disease‐free survival and neoadjuvant chemotherapy response in TNBC but not Her‐2–enriched breast cancer [7, 8]. Estrogen receptor–negative breast cancer with TIL presence responds to anthracycline‐based chemotherapy. CD8^+^ cytotoxic T lymphocytes (CTLs), vital TIL components, synergize with chemotherapeutic agents [9]. Antitumor activity is associated with natural killer cells, CTLs, T helper cells, and T follicular helper cells in the tumor microenvironment [10]. Cancer‐secreted chemokines recruit MDSCs, promoting postcytotoxic treatment survival in cancer cells [11, 12].

Early cancer recurrence biomarkers are crucial. Serum markers, such as carcinoembryonic antigen, cancer antigen 15–3, and cancer antigen 27–29 (also known as mucin 1), are common in breast cancer [13], but their diagnostic and prognostic utility is limited due to low sensitivity and specificity. New biomarkers, including CTCs, microRNAs, tumor antigens, and extracellular vesicles, aid monitoring and early detection of recurrence or metastasis [14]. Liquid biopsy offers diagnostic, predictive, and treatment‐monitoring potential [15], albeit with limited clinical validity. Additionally, assay sensitivity, specificity, and cost must be considered, and establishing tools with heightened sensitivity, ease of use, and affordability is imperative. Because of the importance and convenience of liquid biopsy, other clinical biomarkers are required.

Circulating immune cells hold potential as biomarkers for detecting recurrence or metastasis in patients with cancer. Peripheral granulocytic myeloid cells mirror tumor‐infiltrating immune subtypes in colorectal cancer [16]. Baseline CD4^+^/CD8^+^ ratio predicts prognosis in patients with advanced gastric and esophageal cancer receiving immune checkpoint inhibitor treatment [17]. Regarding non‐small cell lung cancer, responders to immune checkpoint inhibitors exhibit elevated circulating CD4^+^ and CD8^+^ T‐cell proportions [18]. Robust tumor‐circulating immune cell abundance and interactions benefit patients with advanced gastrointestinal cancer considered responders, distinct from those considered progressors [19]. Diminished circulating CD3^−^CD56^+^ natural killer cell levels predict shorter overall survival and progression‐free survival in patients with advanced non‐small cell lung cancer [20]. Fewer circulating CD4^+^PD‐L1^+^ and CD8^+^PD‐L1^+^ T cells correlate with improved progression‐free survival in patients with renal cell carcinoma receiving immune checkpoint inhibitors [21]. The mRNA levels of immune regulators in PBMCs predict disease status of breast cancer, including PD‐L1, FOXP3, CD80, CD40, and CD14 [22]. Based on these previous findings, we hypothesize that circulating immune cells potentially indicate breast cancer status. In the present study, we aim to explore T lymphocyte and MDSC subtypes in this context.

## 2 Materials and Methods

### 2.1 Patients and Blood Samples

In total, 19 healthy controls and 27 patients with primary breast cancer were enrolled at National Cheng Kung University Hospital between September 2017 and July 2018. The patients with other malignancy, younger than 18 years old, or receiving neoadjuvant chemotherapy were excluded. The breast cancer group were subdivided into testing and validation groups (Figure S1). Healthy controls underwent a single blood collection for flow cytometry analysis. In the breast cancer group, preoperative blood sampling occurred before radical operation and standard adjuvant therapy. Postoperative blood sampling was conducted 1 year after therapy completion (adjuvant chemotherapy, radiotherapy, and anti‐Her‐2 therapy) during the phase of adjuvant endocrine therapy as needed. In a randomly selected validation group, one patient with stage IV breast cancer and bone metastases received antiestrogen therapy for bone metastases control followed by modified radical mastectomy. The second blood sampling was performed 1 year post‐operation during the partial response phase. Another patient in the validation group with stage IIB cancer experienced recurrence 1 year post‐operation, with the second blood sampling performed during recurrence evaluation.

For each blood collection, 9 mL of fresh blood was obtained, and 3‐ml‐samples were analyzed for complete blood count. Neutrophil‐to‐lymphocyte ratio (NLR) was derived from neutrophil and lymphocyte percentages. In addition, other 6 mL of fresh blood mixed with Ficoll–Paque PLUS (Amersham Biosciences, Piscataway, NJ, USA) underwent centrifugation at 790 *g* for 20 min. After centrifugation, supernatant plasma was collected for enzyme‐linked immunosorbent assay (ELISA). Lysis buffer (Sigma–Aldrich, Burlington, MA, USA) was added into deposit to lyse red blood cells, purifying whole blood cells for flow cytometry (FACScan, Becton–Dickinson, Franklin Lakes, NJ, USA).

### 2.2 Orthotopic Breast Cancer Model in Immunocompetent Mice

The TNBC cell line PY8119 was obtained from ATCC. C57BL/6 mice received 2.5 × 10^5^ cancer cells, injected into the right third mammary fat pad post anesthesia on Day 1. Tumor detection occurred around 6–7 days postinjection, with thrice‐weekly tracking of tumor size, mouse weight, and survival. Tumor size was calculated using the following formula: 0.5 (Length × width^2^). Submandibular plexus puncture was used for blood collection [23]. Flow cytometry was used to identify CD3^+^CD4^+^ T lymphocytes, CTLs (CD3^+^CD8^+^ lymphocytes), activated CTLs (CD3^+^CD8^+^CD107a^+^ cells), polymorphonuclear (PMN)‐MDSCs (CD11b^+^Ly6C^−^Ly6G^+^ cells), and monocytic (M)‐MDSCs (CD11b^+^Ly6C^+^Ly6G^−^ cells). Complete resection of orthotopic breast cancer was performed on Day 22. Blood was collected on Day 0 (healthy mice), Day 8 (tumor‐carrying mice), and Day 28 (tumor‐free mice).

### 2.3 Flow Cytometry

Circulating immune cells were incubated with monoclonal primary antibodies (BD Biosciences, Franklin Lakes, NJ, USA) at 4°C overnight. Double stain with anti‐CD3‐FITC (Fluorescein Isothiocyanate) and anti‐CD4‐PE (Phycoerythrin) antibody was used to identify human/mouse CD3^+^CD4^+^ T lymphocytes. Double stain with anti‐CD3‐FITC and anti‐CD8‐PE antibody was used to identify human/mouse CD3^+^CD8^+^ lymphocytes. MDSCs were divided into PMN‐MDSCs and M‐MDSC, with the former including CD11b^+^CD14^−^ CD33^+^CD15^+^, CD33^+^CD14^−^ (human) or CD11b^+^Ly6C^−^Ly6G^+^ (mouse), and the latter including CD11b^+^CD14^+^, CD33^+^CD15^−^, CD33^+^CD14^+^ (human) or CD11b^+^Ly6C^+^Ly6G^−^ (mouse). Double stain with anti‐CD11b‐PE and anti‐CD14‐FITC was used to identify human CD11b^+^CD14^−^ and CD11b^+^CD14^+^ cells. Double stain with anti‐CD33‐PE and anti‐CD15‐FITC was used to identify human CD33^+^CD15^+^ and CD33^+^CD15^−^ cells. Double stain with anti‐CD33‐PE and anti‐CD14‐FITC was used to identify human CD33^+^CD14^−^ and CD33^+^CD14^+^ cells. Triple stain with anti‐CD3‐FITC, anti‐CD8‐PE, and anti‐CD107a‐Cy7 (Cyanine 7) antibody was used to identify mouse CD3^+^CD8^+^CD107a^+^ activated CTLs. Triple stain with anti‐CD11b‐PE, anti‐Ly6C‐BV421 (Brilliant Violet 421), and anti‐Ly6G‐FITC was used to identify mouse CD11b^+^Ly6C^−^Ly6G^+^ and CD11b^+^Ly6C^+^Ly6G^−^ cells. Staining buffer (1 mL, BD Biosciences) was added before flow cytometry analysis. Data analysis was performed via WinMDI 2.9.

### 2.4 Enzyme‐Linked Immunosorbent Assay (ELISA)

Plasma samples were acquired from patients with breast cancer and healthy volunteers who gave appropriate informed consent under Institutional Review Board approval. An enzyme‐linked immunosorbent assay was performed using a commercial kit (R&D Systems, Minneapolis, MN, USA) following manufacturer instructions. Mouse anti‐human‐CCL2 capture antibody was added to wells and incubated overnight for coating on plate. After delicate washing, 100 μL standard recombinant hCCL2 or suitable plasma samples were added and incubated 2 h at room temperature. Subsequently, biotinylated goat anti‐human‐CCL2 detection antibody was incubated for 2 h at room temperature, followed by the addition of streptavidin‐horseradish peroxidase. After a 20‐min incubation, a stop solution was added to end the color reaction, with absorbance subsequently being read at 540 nm.

### 2.5 Statistical Analysis

STATA version 16.1 (StataCorp, College Station, TX, USA) was used to perform all statistical analyses. Categorical variable univariate analysis was performed using the chi‐squared test, whereas non‐normally distributed continuous variables were compared using the Mann–Whitney test (for 2 groups) or Kruskal–Wallis test (for > 2 groups). Paired preoperative and postoperative data in patients or mice were compared using the Wilcoxon matched‐pairs signed‐ranks one‐sided test. Significance was defined as *p* < 0.05.

## 3 Results

### 3.1 Circulating Immune Cells in Patients With Breast Cancer Studying by Flow Cytometry

The median age was 40 years in healthy controls (range: 28–50 years), and 52 years in breast cancer patients (range: 29–73 years). Table 1 shows demographics and pathological findings of patients in the breast cancer group, who all underwent standard treatment and follow‐up at our hospital. Simultaneous complete blood counting of white blood cells and flow cytometry of specific cell types were performed. In the testing group comprising 22 patients with breast cancer, lymphocyte and neutrophil counts were recorded preoperatively. NLR was compared between T1 and T2/T3 cases, grade I/II and grade III cases, lymph node metastasis–negative and lymph node metastasis–positive cases, and different breast cancer subtypes (Figure S2). Immune cell proportions did not correlate with these factors, except grade III breast cancer correlating with a higher preoperative NLR (Figure S2B).

**TABLE 1:** Demographics and pathological diagnosis of patients with breast cancer.

	Testing group (*N* = 22)	Validation group (*N* = 5)
Age, years, median (range)	52 (38–60)	51 (29–73)
Operation method		
MRM	10 (45%)	2 (40%)
TM + SLNB	4 (18%)	3 (60%)
BCS + ALND	1 (5%)	0
BCS + SLNB	7 (32%)	0
Tumor size, cm, median (range)	2.1 (0.1–7.0)	1.5 (0.1–6.5)
Tumor stage		
T1	10 (45%)	4 (80%)
T2	11 (50%)	1 (20%)
T3	1 (5%)	0
Histological differentiation		
Grade I	1 (5%)	0
Grade II	14 (63%)	2 (40%)
Grade III	7 (32%)	3 (60%)
Extensive intraductal components		
Negative	17 (77%)	2 (40%)
Positive	5 (23%)	3 (60%)
Number of positive lymph node, median (range)	1 (0–26)	0 (0–2)
Number of total lymph node, median (range)	11 (2–32)	5 (2–20)
Lymphatic tumor emboli		
Negative	15 (68%)	3 (60%)
Positive	7 (32%)	2 (40%)
Nipple invasion		
Negative	10 (72%)	5 (100%)
Positive	4 (29%)	0
Lymph node metastasis		
Negative	10 (45%)	3 (60%)
Positive	12 (55%)	2 (40%)
Nodal stage		
N0	10 (45%)	3 (60%)
N1	5 (23%)	2 (40%)
N2	5 (23%)	0
N3	2 (9%)	0
Extranodal extension		
Negative	15 (68%)	5 (100%)
Positive	7 (32%)	0
Estrogen receptor		
Negative	5 (23%)	2 (40%)
Positive	17 (77%)	3 (60%)
HER2 receptor		
Negative	18 (82%)	4 (80%)
Positive	4 (18%)	1 (20%)
TNM Stage		
Stage I	6 (27%)	2 (40%)
Stage II	9 (41%)	2 (40%)
Stage III	7 (32%)	1 (20%)
Stage IV	0	
Subtypes		
HR^+^, HER2^−^	16 (73%)	3 (60%)
HR^−^, HER2^+^	4 (18%)	1 (20%)
Triple‐negative breast cancer	2 (9%)	1 (20%)

Abbreviations: ALND, axillary lymph node dissection; BCS, breast‐conserving surgery; HER2, human epidermal growth factor type II receptor; HR, hormone receptor; MRM, modified radical mastectomy; SLNB, sentinel lymph node biopsy; TM, total mastectomy.

Preoperative white blood cell levels exceeded postoperative levels in most patients (Figure S3A). Neutrophil and lymphocyte counts, as well as NLR, remained unchanged before and after radical operation (Figure S3B–D).

### 3.2 Association With Circulating Immune Cells in Flow Cytometry by Pathological Factors

Flow cytometry was used to identify surface markers of specific immune cells in the breast cancer testing group. Each kind of MDSCs were represented by three types of surface markers. PMN‐MDSCs (CD11b^+^CD14^−^, CD33^+^CD15^+^, and CD33^+^CD14^−^) were correlated with each other (Figure S4A). Expression pattern of CD11b^+^CD14^+^, CD33^+^CD15^−^, and CD33^+^CD14^+^ (markers of M‐MDSCs) were similar (Figure S4B). Correlations among different immune cell subtypes were determined (Figure S5), revealing opposing expression levels of PMN‐MDSCs and M‐MDSCs.

**FIGURE 1:**
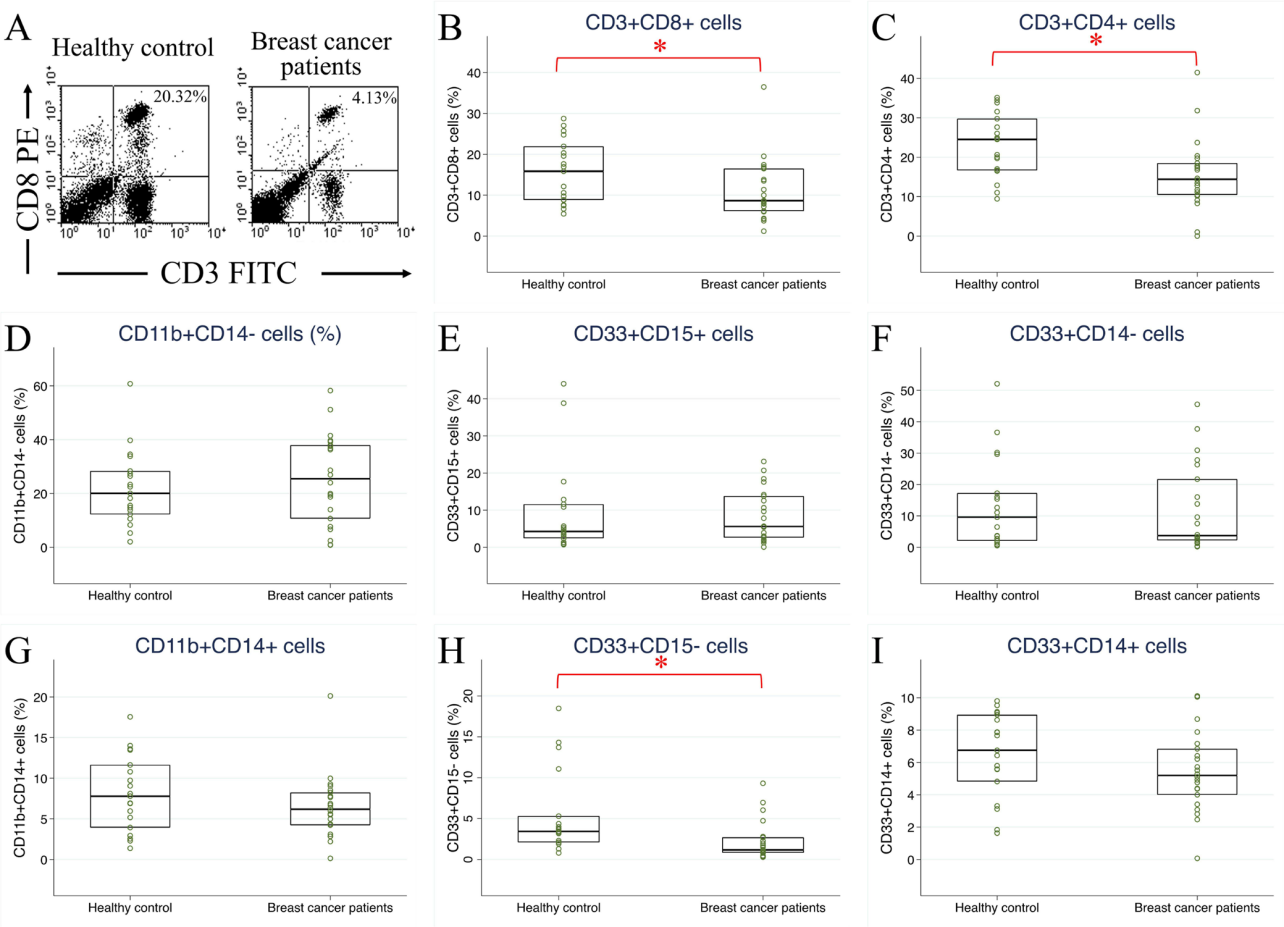
Flow cytometry analysis of circulating immune cells in healthy controls and preoperative patients with breast cancer in the testing group. (A) Example of double staining with CD3 and CD8 in flow cytometry. (B) CD3^+^CD8^+^ (*p* = 0.038), (C) CD3^+^CD4^+^ (*p* = 0.007), (D) CD11b^+^CD14^−^ (*p* = 0.448), (E) CD33^+^CD15^+^ (*p* = 0.610), (F) CD33^+^CD14^−^ (*p* = 0.676), (G) CD11b^+^CD14^+^ (*p* = 0.214), (H) CD33^+^CD15^−^ (*p* = 0.002), and (I) CD33^+^CD14^+^ (*p* = 0.147) cell populations. *p < 0.05.

Comparisons of circulating immune cells from blood were made between healthy controls and patients with breast cancer preoperatively in the testing group. The median and range of cell percentages are shown in Table S1. CD3^+^CD8^+^ cell proportions were lower in patients with breast cancer than in healthy controls (Figure 1A,B, *p* = 0.038). Additionally, CD3^+^CD4^+^ T lymphocyte and M‐MDSC levels were higher in healthy controls (Figure 1C,G–I, *p* = 0.007). Levels of CD33^+^CD15^−^, an M‐MDSC subtype, were decreased in the breast cancer group (Figure 1H, *p* = 0.002). The expression of PMN‐MDSCs differed among the three subtypes (Figure 1D–F). Patients with breast cancer exhibited lower CD3^+^CD4^+^ T lymphocyte, CD3^+^CD8^+^ CTL, and CD33^+^CD15^−^ M‐MDSC levels compared with healthy controls.

**FIGURE 2:**
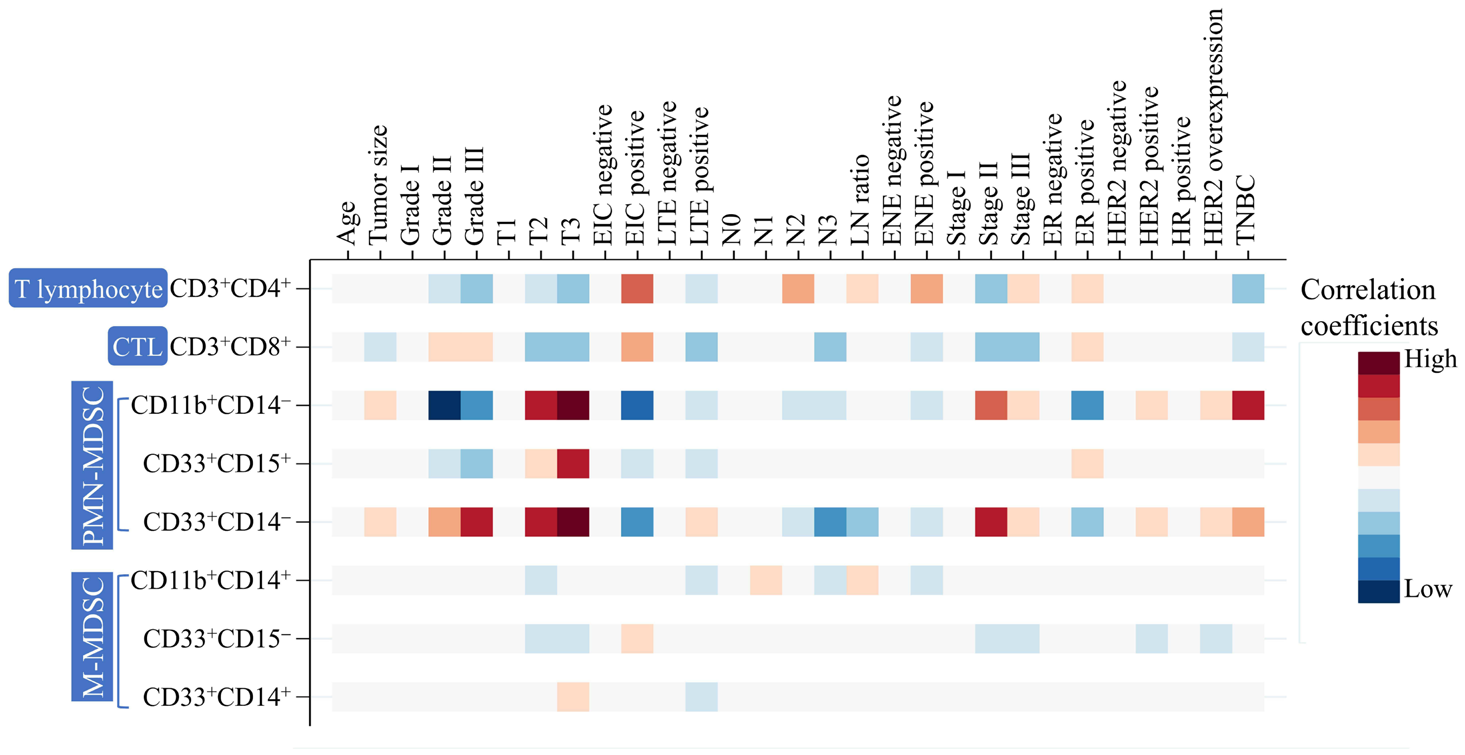
Heat map illustrating correlations between pathological factors and preoperative circulating immune cells. Red squares denote pathological factors with strong positive correlation coefficients, blue squares indicate strong negative correlations, and white squares indicate no correlation with circulating immune cells.

In 22 patients in the breast cancer testing group, preoperative immune cells were correlated with pathological factors (Figure 2). CD3^+^CD8^+^ CTLs were correlated with higher cancer grades (grade II and III) and positive extensive intraductal components. CD3^+^CD4^+^ T lymphocytes were correlated with positive extensive intraductal components, nodal stage N2 cancer, and positive extranodal extension of lymph node metastasis. PMN‐MDSCs, especially CD11b^+^CD14^−^ and CD33^+^CD14^−^ cells, were correlated with advanced tumor stage (T2 and T3), cancer stage (stage II and III), and the TNBC subtype. Other correlations were weak owing to the limited number of patients.

**FIGURE 3:**
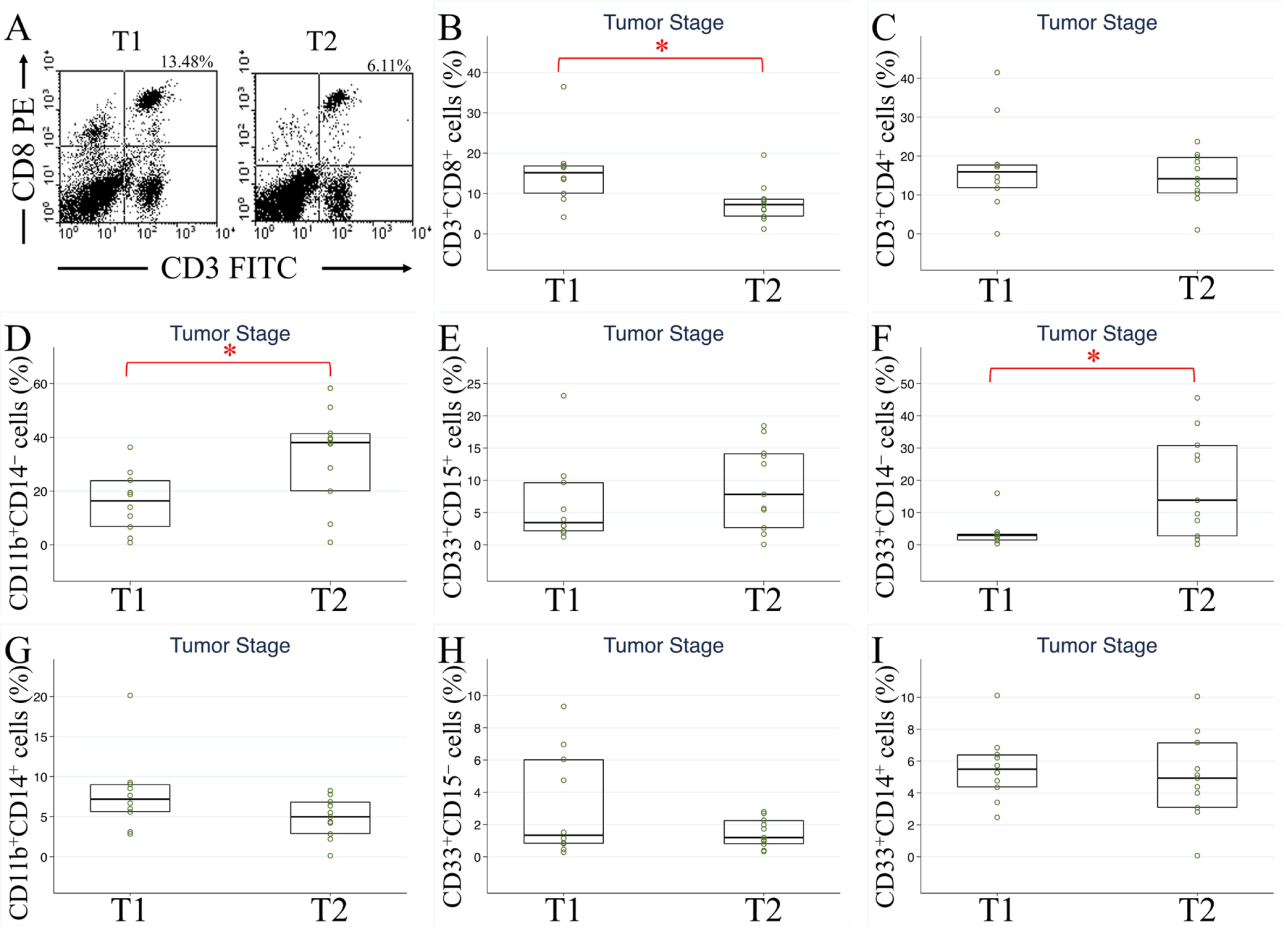
Correlation of circulating immune cells with tumor stage in the breast cancer testing group. (A) Example of double staining with CD3 and CD8 in flow cytometry. (B) CD3^+^CD8^+^ (*p* = 0.013), (C) CD3^+^CD4^+^ (*p* = 0.778), (D) CD11b^+^CD14^−^ (*p* = 0.013), (E) CD33^+^CD15^+^ (*p* = 0.349), (F) CD33^+^CD14^−^ (*p* = 0.036), (G) CD11b^+^CD14^+^ (*p* = 0.072), (H) CD33^+^CD15^−^ (*p* = 0.605), and (I) CD33^+^CD14^+^ (*p* = 0.605) cell populations. *p < 0.05.

Large tumor size is a predictor of poor prognosis in patients with breast cancer [24]. There were 10 patients with T1 and 11 ones with T2 tumor stage in testing group. Circulating immune cells were compared between T1 and T2 cases, with T2 cases being associated with decreased CD3^+^CD8^+^ cell proportions (*p* = 0.013) and increased CD11b^+^CD14^−^ (*p* = 0.013)/CD33^+^CD14^−^ (*p* = 0.036) PMN‐MDSC levels compared with T1 cases (Figure 3A,B,D,F). Other immune cells were not correlated with tumor stage (Figure 3C,E,G–I). Additionally, larger tumors exhibited fewer CTLs and more PMN‐MDSCs.

Detection of lymphatic tumor emboli is a predictor of poor survival in patients with breast cancer [25]. Seven patients in the testing group exhibited lymphatic tumor emboli in primary cancer, and these patients displayed decreased CD3^+^CD8^+^ cells (Figure S6A,B) and increased CD11b^+^CD14^+^/CD33^+^CD14^+^ M‐MDSC levels (Figure S6G,I). Other immune cells were not correlated with tumor stage (Figure S6C–F,J). Patients with positive lymphatic tumor emboli also had lower levels of circulating CD3^+^CD8^+^ CTLs and M‐MDSCs.

### 3.3 Comparison Circulating Immune Cells Between Preoperative and Postoperative Conditions

**FIGURE 4:**
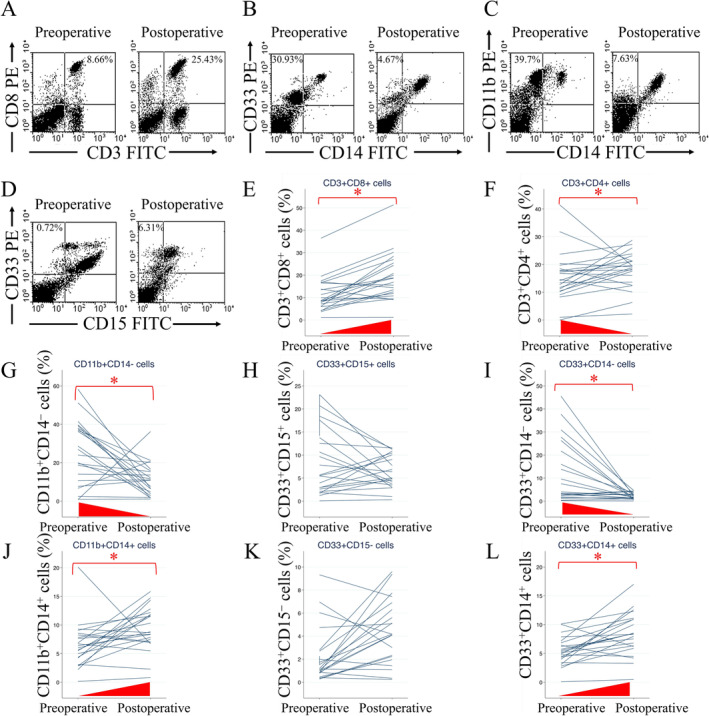
Comparison of circulating immune cells before and after surgery and standard treatment. (A) Examples of double staining with CD3 and CD8, (B) CD14 and CD33, (C) CD14 and CD11b, (D) CD15 and CD33 cells in flow cytometry. (E) CD3^+^CD8^+^ (*p* = 0.002), (F) CD3^+^CD4^+^ (*p* = 0.026), (G) CD11b^+^CD14^−^ (*p* = 0.009), (H) CD33^+^CD15^+^ (*p* = 0.500), (I) CD33^+^CD14^−^ (*p* = 0.009), (J) CD11b^+^CD14^+^ (*p* = 0.026), (K) CD33^+^CD15^−^ (*p* = 0.095), and (L) CD33^+^CD14^+^ (*p* = 0.002) cell population. *p < 0.05.

Postoperative circulating immune cells from the breast cancer testing group were compared with preoperative data to assess recovery after standard treatment (Figure 4). Elevated proportions of postoperative CD3^+^CD8^+^ (*p* = 0.002), CD11b^+^CD14^+^ (*p* = 0.026), and CD33^+^CD14^+^ (*p* = 0.002) cells were detected compared with preoperative levels (Figure 4A–C,E,J–L). No consistent pattern of change was observed in CD33^+^CD15^+^ and CD33^+^CD15^−^ cells (Figure 4D,H,K). Decreased proportions of postoperative CD3^+^CD4^+^ (*p* = 0.026), CD11b^+^CD14^−^ (*p* = 0.009), and CD33^+^CD14^−^ (*p* = 0.009) cells were detected (Figure 4F,G,I). These results indicate an increase in CD3^+^CD8^+^ CTL/CD11b^+^CD14^+^ and CD33^+^CD14^+^ M‐MDSC levels, as well as a decrease in CD3^+^CD4^+^ T lymphocyte/PMN‐MDSC levels, following treatment.

Change patterns of circulating immune cells from preoperative to postoperative conditions were comparable between patients with or without lymphatic tumor emboli (Figure S7). Patients with lymphatic tumor emboli exhibited elevated CD3^+^CD8^+^ cells (Figure S7A,B). CD3^+^CD4^+^, CD33^+^CD15^+^, CD11b^+^CD14^+^, and CD33^+^CD15^−^ cells exhibited no consistent pattern of change (Figure S7C,E,G,H). Increased proportions of postoperative CD33^+^CD14^+^ cells relative to preoperative levels were evident in patients without lymphatic tumor emboli (Figure S7I). Conversely, patients without lymphatic tumor emboli showed increased proportions of postoperative CD11b^+^CD14^−^ and CD33^+^CD14^−^ cells (Figure S7D,F). These findings reinforced the trend of increased CTL and M‐MDSC levels and decreased PMN‐MDSC levels posttreatment.

In the validation group, one patient with breast cancer experienced recurrence, leading to decreased levels of circulating CD3^+^CD8^+^ cells with disease progression (Figure S8A). Patients showing partial response (Figure S8B) or complete response (Figure S8C) maintained stable CD3^+^CD8^+^ cell levels. Notably, NLR failed to accurately represent disease status.

### 3.4 Plasma Level of Regulatory Cytokines Studying by ELISA

**FIGURE 5:**
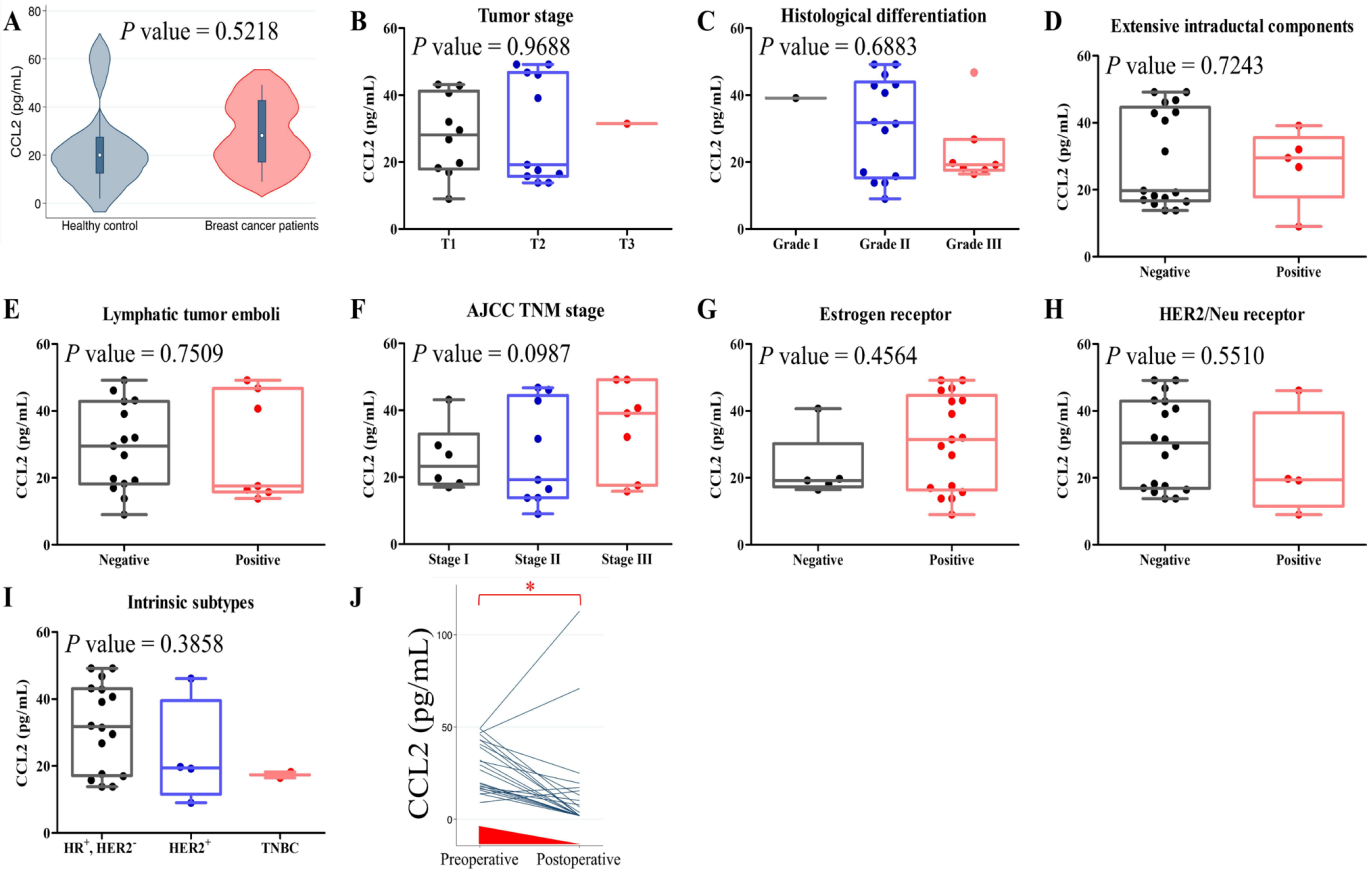
Plasma levels of CCL2 in study subjects. (A) Healthy controls vs. patients with breast cancer. Comparisons (B) among different tumor stages, (C) among different grades, (D) between negative and positive extensive intraductal components, (E) between negative and positive lymphatic tumor emboli, (F) among different cancer stages, (G) between negative and positive estrogen receptor status, (H) between negative and positive Her‐2/Neu receptor status, (I) among different subtypes, and (J) between preoperative and postoperative levels. The nonparametric Mann–Whitney test was used for statistical analysis in A−I, whereas the nonparametric Wilcoxon matched‐pairs signed‐ranks one‐sided test was used in J (*p < 0.05).

Cytokines play a pivotal role in regulating circulating immune cells, and CCL2 is known to recruit macrophages and immunosuppressive cells to the tumor microenvironment [26]. The median plasma CCL2 level was 19.99 pg/mL (range: 1.95–64.87 pg/mL) in healthy controls and 28.15 pg/mL (range: 9.01–49.15 pg/mL) in the breast cancer testing group (Figure 5A). Plasma CCL2 levels were not correlated with pathological factors or subtypes of breast cancer (Figure 5B–I). However, most patients exhibited a reduction in plasma CCL2 levels after curative surgery (Figure 5J), suggesting that individuals with breast cancer tended to exhibit higher plasma CCL2 levels.

### 3.5 Orthotopic Model of Breast Cancer in Immunocompetent Mice

**FIGURE 6:**
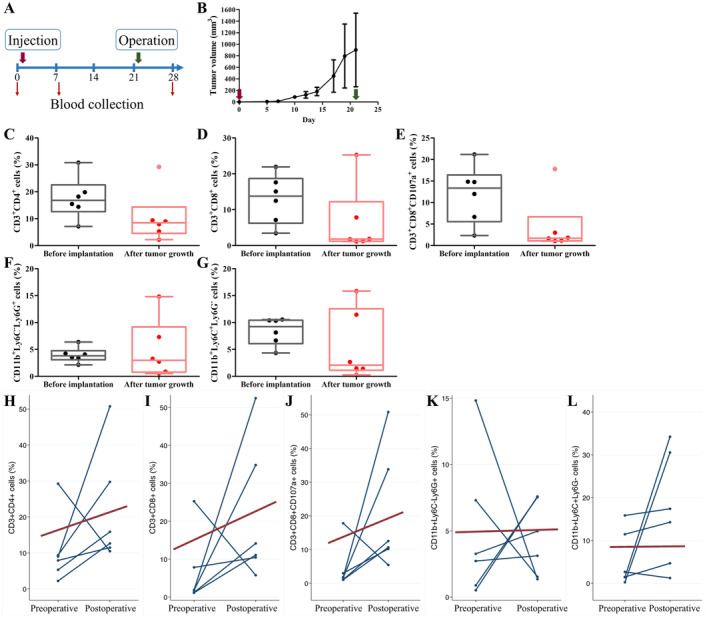
Circulating immune cells in mice with orthotopic breast cancer (*n* = 5). (A) Study design of orthotopic breast cancer in mice. (B) Tumor size of orthotopic breast cancer. (C–G) Comparison between healthy mice (Day 0, before implantation of mouse breast cancer cells) and tumor‐carrying mice (Day 8, after tumor growth). (C) CD3^+^CD4^+^ (*p* = 0.109), (D) CD3^+^CD8^+^ (*p* = 0.109), (E) CD3^+^CD8^+^CD107a^+^ (*p* = 0.055), (F) CD11b^+^Ly6C^−^Ly6G^+^ (*p* = 0.522), and (G) CD11b^+^Ly6C^+^Ly6G^−^ (*p* = 0.337) cell populations. The nonparametric Mann–Whitney test was used for statistical analysis. (H–L) Comparison between tumor‐carrying mice (Day 8, after tumor growth) and tumor‐free mice (Day 28, after cancer resection). (H) CD3^+^CD4^+^ (*p* = 0.109), (I) CD3^+^CD8^+^ (*p* = 0.109), (J) CD3^+^CD8^+^CD107a^+^ (*p* = 0.109), (K) CD11b^+^Ly6C^−^Ly6G^+^ (*p* = 0.344), and (L) CD11b^+^Ly6C^+^Ly6G^−^ (*p* = 0.109) cell populations. The nonparametric Wilcoxon matched‐pairs signed‐ranks one‐sided test was used for statistical analysis.

An orthotopic breast cancer model was established for validating finding from patients by injecting PY8119 breast cancer cells into immunocompetent C57BL/6 mice. Circulating immune cells on Day 0 represented the state of healthy mice prior to cancer cell implantation. Subsequent to the injection of mouse breast cancer cells, orthotopic tumors developed. Circulating immune cells on Day 8 reflected tumor‐bearing mice after tumor growth. After surgical resection of the tumor on Day 22, blood was collected on Day 28 to determine mice that were tumor‐free postoperatively. The study design is depicted in Figure 6A, and tumor size is illustrated in Figure 6B. CD3^+^CD8^+^ CTLs, CD3^+^CD4^+^ T lymphocytes, and CD3^+^CD8^+^CD107a^+^ activated CTLs were more abundant in healthy mice before implantation compared with tumor‐bearing mice after tumor growth (Figure 6C–E). In contrast, CD11b^+^Ly6C^−^Ly6G^+^ PMN‐MDSCs and CD11b^+^Ly6C^+^Ly6G^−^ M‐MDSCs showed similar levels before and after tumor growth (Figure 6F,G). Postoperative levels (Day 28) of circulating immune cells were compared with preoperative levels (Day 8): CD3^+^CD8^+^ CTL, CD3^+^CD4^+^ T lymphocyte, and CD3^+^CD8^+^CD107a^+^ activated CTL levels increased postoperatively (Figure 6H–J), whereas CD11b^+^Ly6C^−^Ly6G^+^ PMN‐MDSC and CD11b^+^Ly6C^+^Ly6G^−^ M‐MDSC levels remained steady (Figure 6I,K). When combined with patient data, the level of circulating CTLs emerged as a potential prognostic marker for breast cancer.

## 4 Discussion

In this study, our objective was to identify potential biomarkers for breast cancer by investigating circulating T lymphocytes, CTLs, and MDSCs across various stages of breast cancer and in an orthotopic breast cancer model. We found that circulating CD3^+^CD8^+^ CTL levels decreased in patients with breast cancer, increased after treatment, and decreased again upon recurrence. Thus, plasma levels of CD3^+^CD8^+^ CTLs hold promise as potential biomarkers reflecting disease status in breast cancer.

**FIGURE 7:**
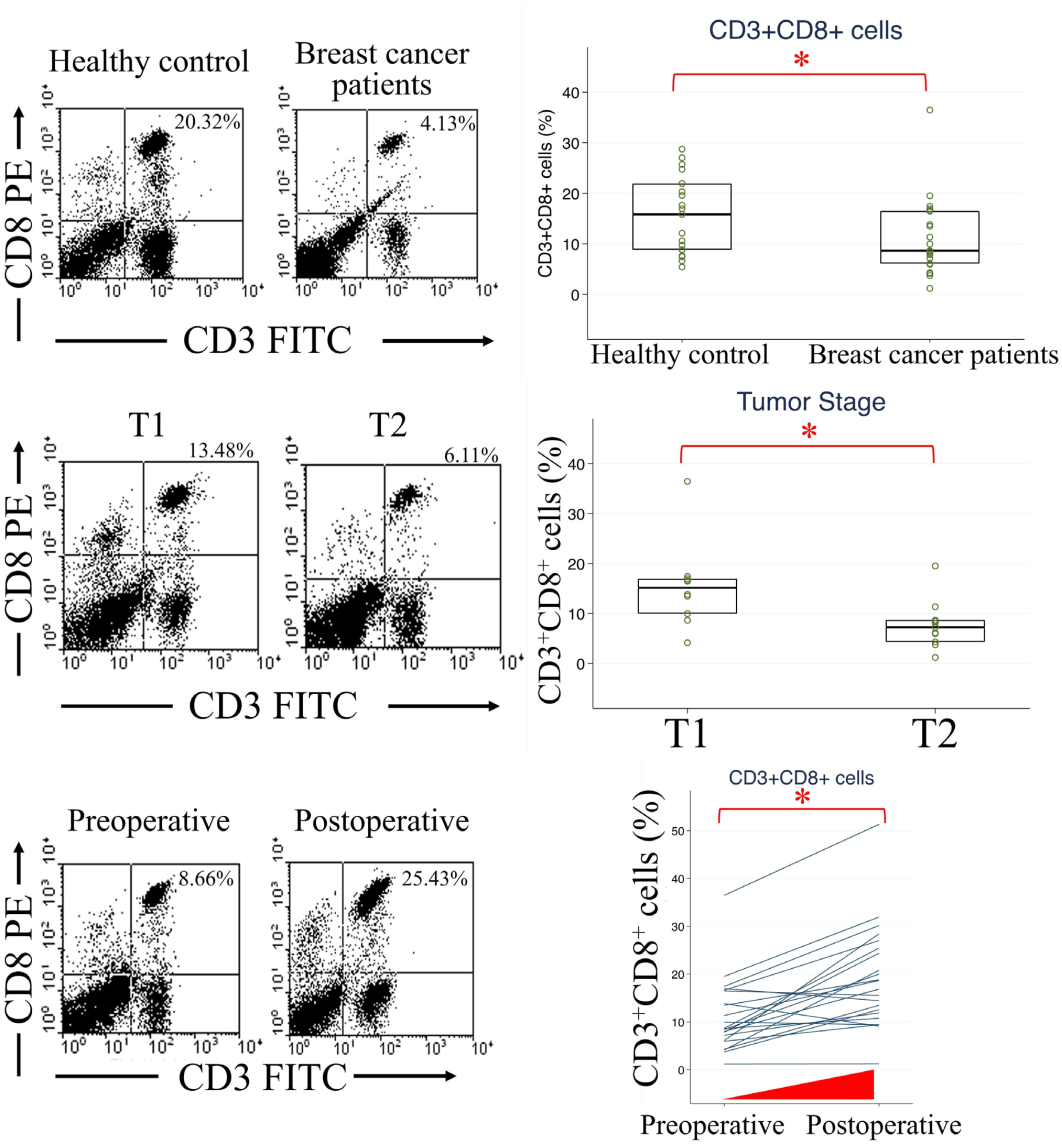
Graphical abstracts. Results from flow cytometry analyses of 27 breast cancer patients and 19 healthy controls to evaluate circulating CD3^+^CD8^+^ cytotoxic T cells, comparing between patients versus controls, T1 versus T2, and preoperative versus postoperative were summarized. Circulating CD3^+^CD8^+^ cell levels were lower in breast cancer patients, T2 cancer patients, and elevated posttreatment.

TILs are carried through the tributaries of the axillary vein, and circulating immune cells may mirror the composition of tumor‐infiltrating immune cells in breast cancer [27]. The proportions of specific lymphocyte subtypes differ among patients with cancer. In non‐small cell lung cancer patients, levels of T lymphocytes, natural killer cells, CD8^+^ T cells, and CD4^+^ T cells are diminished compared with those in healthy individuals [28]. Additionally, elevated circulating PMN‐MDSC levels are associated with poorer recurrence‐free survival of patients with hepatocellular carcinoma [29]. Colorectal cancer–diagnosed patients with higher CD16^+^ natural killer T‐cell counts exhibit shorter disease‐free survival [30]. In patients with hepatocellular carcinoma, an increased number of circulating CD8^+^ T cells enhances the effectiveness of immunotherapy [31]. Notably, in patients with breast cancer, CD117^+^ granulocytes correlate with cancer status, and CD45RO^+^ CD4^+^ memory T‐cell counts rise after adjuvant radiotherapy [32]. Using flow cytometry, we analyzed circulating immune cells. Our findings indicate that the level of CD3^+^CD8^+^ CTLs is lower in preoperative patients with breast cancer than in healthy individuals and inversely correlates with tumor stage, positive lymphatic tumor emboli, and nodal stage N3. Following treatment, circulating CD3^+^CD8^+^ CTL levels increase, whereas patients exhibiting disease progression show decreased CD3^+^CD8^+^ CTL levels (Figure 7). We observed a similar trend in a mouse model of breast cancer. Levels of CD3^+^CD8^+^ CTLs and activated CTLs are higher in healthy mice (Day 0, before implantation of mouse breast cancer cells), decreased in tumor‐carrying mice (Day 8, after tumor growth), then returned to higher level after resection of cancer (Day 28). Therefore, our study confirms circulating CD3^+^CD8^+^ CTLs as a valuable prognostic biomarker for breast cancer.

MDSCs play a pivotal role on immune suppression in breast cancer by generating reactive oxygen species, arginase, and cytokines that hinder the activity of other immune cells, including T cells, dendritic cells, and natural killer cells [33]. Their presence in peripheral blood or tumors is linked to cancer stage, grade, and patient survival. Furthermore, MDSCs are involved in regulating cancer dissemination [34]. Distinct surface markers distinguish between PMN‐MDSCs and M‐MDSCs [35]. M‐MDSCs promote the acquisition of a mesenchymal phenotype and stemness characteristics, facilitating cancer dissemination from the primary site. Conversely, PMN‐MDSCs promote metastatic growth by reverting the epithelial phenotype of already disseminated tumor cells [36]. We used CD11b^+^CD14^−^, CD33^+^CD15^+^, and CD33^+^CD14^−^ to identify human PMN‐MDSCs and CD11b^+^CD14^+^, CD33^+^CD15^−^, and CD33^+^CD14^+^ for human M‐MDSCs, and we observed strong correlations among these markers within each group. However, PMN‐MDSCs and M‐MDSCs exhibited contrasting expression patterns. Patients with breast cancer displayed elevated levels of CD33^+^CD15^−^ M‐MDSCs compared with healthy controls. Additionally, CD33^+^CD14^−^ PMN‐MDSC levels increased in patients with larger tumors, whereas CD11b^+^CD14^+^/CD33^+^CD14^+^ M‐MDSC levels decreased in those with lymphatic tumor emboli. Posttreatment, CD11b^+^CD14^−^/CD33^+^CD14^−^ PMN‐MDSC levels decreased, whereas CD11b^+^CD14^+^/ CD33^+^CD14^+^ M‐MDSC levels increased. The opposing expression pattern of PMN‐MDSCs and M‐MDSCs suggests that low PMN‐MDSC and high M‐MDSC levels could potentially serve as biomarkers for a cancer‐free status.

The intricate interplay between cancer and immune cells depends on secretory cytokines. CCL2, binding to its receptor C–C chemokine receptor type 2 (CCR2), acts as a chemokine, attracting monocytes during cancer progression. CCL2/CCR2 signaling also promotes early breast tumorigenesis and invasive properties [37]. Specifically, CCL2 triggers phosphorylation of MET receptor tyrosine kinases, inducing proliferation, migration, and glycolysis in breast cancer cells [38]. Blocking CCL2 activates CD8^+^ CTLs, leading to reduced tumorigenesis of lung cancer [39]. Secreted into the tumor microenvironment, CCL2 promotes angiogenesis, as well as the recruitment of tumor‐associated macrophages and MDSCs in breast cancer [40], and plasma CCL2 levels are higher in patients with breast cancer compared with healthy individuals [41]. Elevated CCL2 expression in breast cancer cells is associated with early recurrence [42]. Propagermanium, a CCL2 inhibitor, has shown promising results, downregulating serum IL‐6 levels in a phase I clinical trial, as well as demonstrating clinical safety and holding potential as an antimetastatic agent in breast cancer treatment [43]. Our study confirms elevated CCL2 levels exist in patients with breast cancer relative to healthy controls with large overlapping CCL2. Additionally, although CCL2 was not correlated with pathological factors or cancer subtypes, it exhibited decreased levels posttreatment, suggesting that it is a potential prognostic marker for breast cancer.

NLR has been used as a prognostic indicator in previous studies for solid tumor [44], and elevated NLR is associated with advanced breast cancer and poorer patient survival rates [45, 46]. However, NLR cutoff values have varied across studies of cancer, ranging from 2.96 to 5.7 [44, 45, 46, 47]. In our study, patients with grade III breast cancer exhibited a lower NLR, whereas NLR was not correlated with other pathological factors. Despite its accessibility and cost‐effectiveness, the inconsistent findings surrounding NLR have rendered its clinical application challenging.

A major limitation of the present study arises from the relatively small patient cohort. Considerable interindividual variation exists in certain types of circulating immune cells, including CD33^+^CD15^+^ and CD33^+^CD15^−^ cells. In an effort to assess changes in circulating immune cells, we employed a murine model. Interestingly, both human patients with breast cancer and the orthotopic breast cancer mouse model displayed a congruent dynamic pattern in circulating CD3^+^CD8^+^ CTL levels. Conversely, changes in MDSCs were inconsistent between the human breast cancer group and murine model. Markers for human and mouse MDSCs are dissimilar. Human M‐MDSCs are described as HLA‐DR^−^CD11b^+^CD14^+^CD15^−^CD33^High^ and PMN‐MDSCs as HLA‐DR^−^CD11b^+^CD14^−^CD15^+^CD33^Mild^ cells. Mouse M‐MDSCs are represented as CD11b^+^Ly6C^+^Ly6G^−^ and PMN‐MDSCs as CD11b^+^Ly6C^−^Ly6G^+^ cells [48]. In our study, we used CD11b^+^CD14^+^, CD33^+^CD15^−^, and CD33^+^CD14^+^ to identify human M‐MDSCs, and CD11b^+^CD14^−^, CD33^+^CD15^+^, and CD33^+^CD14^−^ for human PMN‐MDSCs. Dissimilar MDSC markers in two species might yield decreased correlations with disease status in breast cancer.

In conclusion, our study highlights the reduced levels of CD3^+^CD8^+^ CTLs in breast cancer–diagnosed patients, which subsequently increase following treatment. Additionally, we observed elevated CCL2 levels in these patients, which subsequently decreased posttreatment. We conclude that levels of circulating CD3^+^CD8^+^ CTLs represent candidates for prognostic biomarkers and treatment targets in breast cancer. To my best knowledge, this is the first report analyzed circulating immune cells in breast cancer including patients and orthotopic mouse model. Future research should focus on elucidating the complex interplay between immune subsets and their influence on breast cancer progression. Exploring MDSC dynamics, tumor microenvironment interactions, and larger patient cohorts are essential for advancing personalized therapeutic approaches in breast cancer management.

## Author Contributions

**Han‐Kun Chen:** writing – original draft (equal). **Yi‐Ling Chen:** conceptualization (equal), writing – review and editing (equal). **Wei‐Pang Chung:** investigation (equal), writing – review and editing (equal). **Zhu‐Jun Loh:** methodology (equal). **Kuo‐Ting Lee:** methodology (equal). **Hui‐Ping Hsu:** conceptualization (equal), data curation (equal), formal analysis (equal), investigation (equal), methodology (equal), validation (equal), writing – review and editing (equal).

## Ethics Statement

The study was reviewed and monitored by Institutional Review Board of National Cheng Kung University Hospital (B‐ER‐108‐400). All experimental protocols were approved by the IRB and all methods were performed in accordance with relevant guidelines and regulations for Good Clinical Practice.

## Conflicts of Interest

The authors declare no conflicts of interest.

## Supporting information


Data S1.

## Data Availability

The data that support the findings of this study are available from the corresponding author upon reasonable request.
