# Supplementary material for: Circulating CD3 + CD8 + T Lymphocytes as Indicators of Disease Status in Patients With Early Breast Cancer
Source: Cancer Med. 2025 Jan 3;14(1):e70547. doi: 10.1002/cam4.70547 (PMC11696249; doi:10.1002/cam4.70547)
Supplement: Supplementary file 1 — Data S1. [file CAM4-14-e70547-s001.docx]

**Circulating CD3^+^CD8^+^ T lymphocytes as indicators of disease status in patients with early breast cancer**


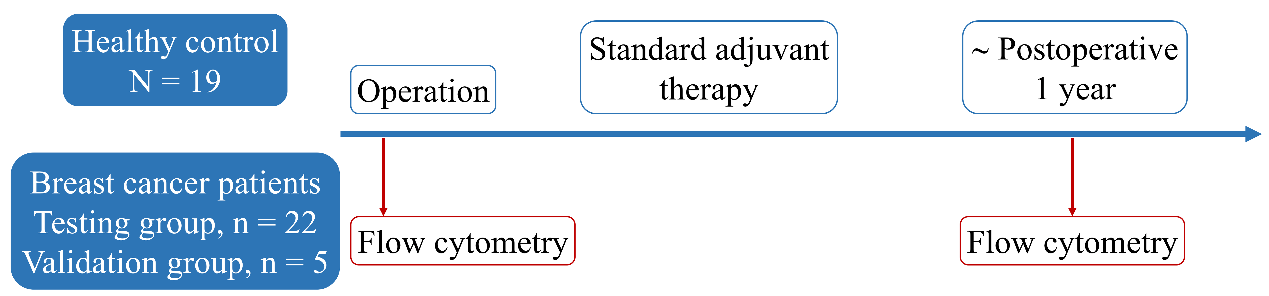


**Supplementary Figure 1.** Study design schematic depicting the enrollment of 19 healthy volunteers and 27 patients with breast cancer.


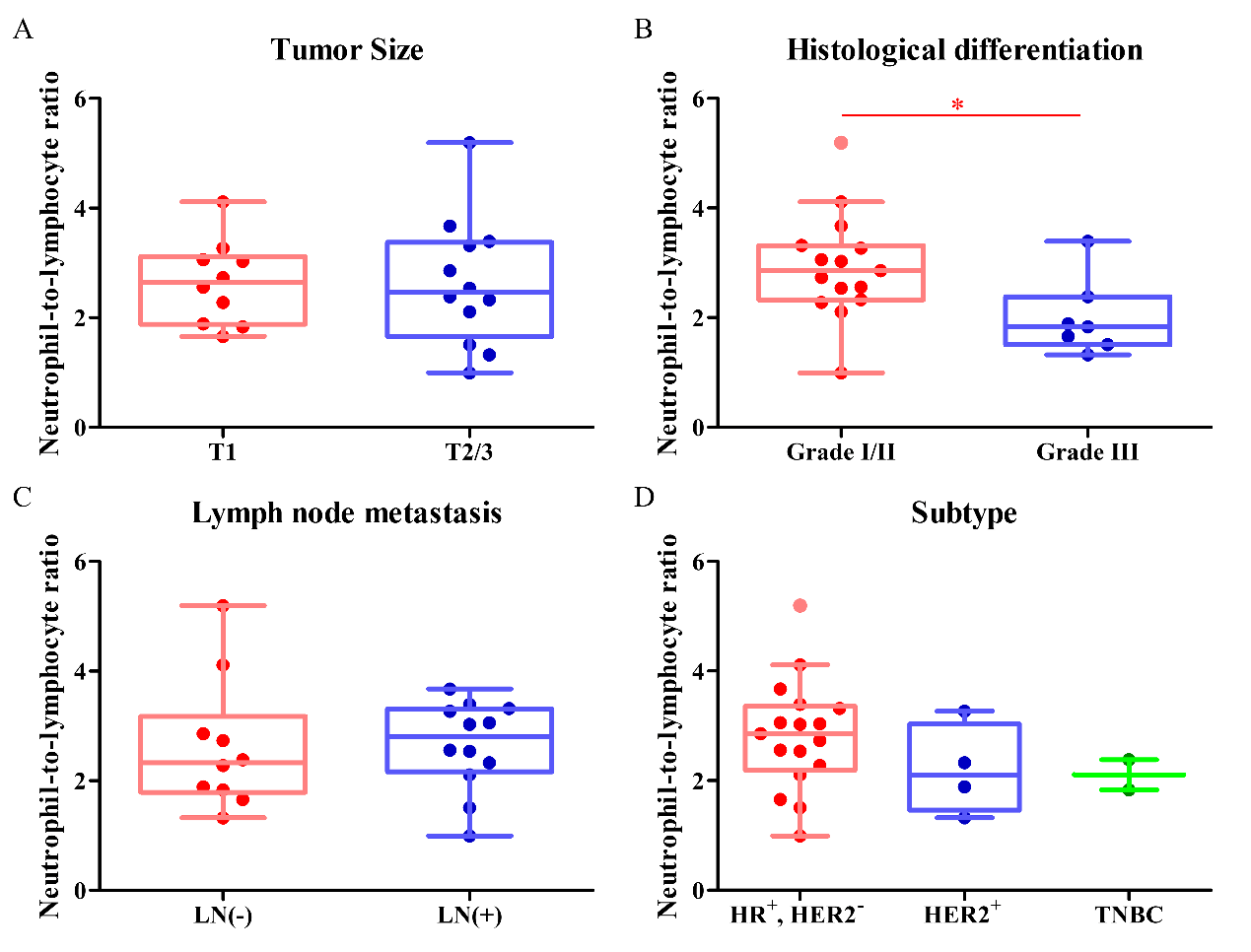


**Supplementary Figure 2.** Neutrophil-to-lymphocyte ratio in the breast cancer testing group. Comparison (A) between T1 and T2/T3 cancers (*P* = 0.895), (B) across different histological differentiation grades (*P* = 0.026), (C) between patients with and without lymph node metastasis (*P* = 0.510), and (D) among different breast cancer subtypes (*P* = 0.332). Abbreviations: HER2, human epidermal growth factor receptor type II; HR, hormone receptor; LN, lymph node; TNBC, triple-negative breast cancer.


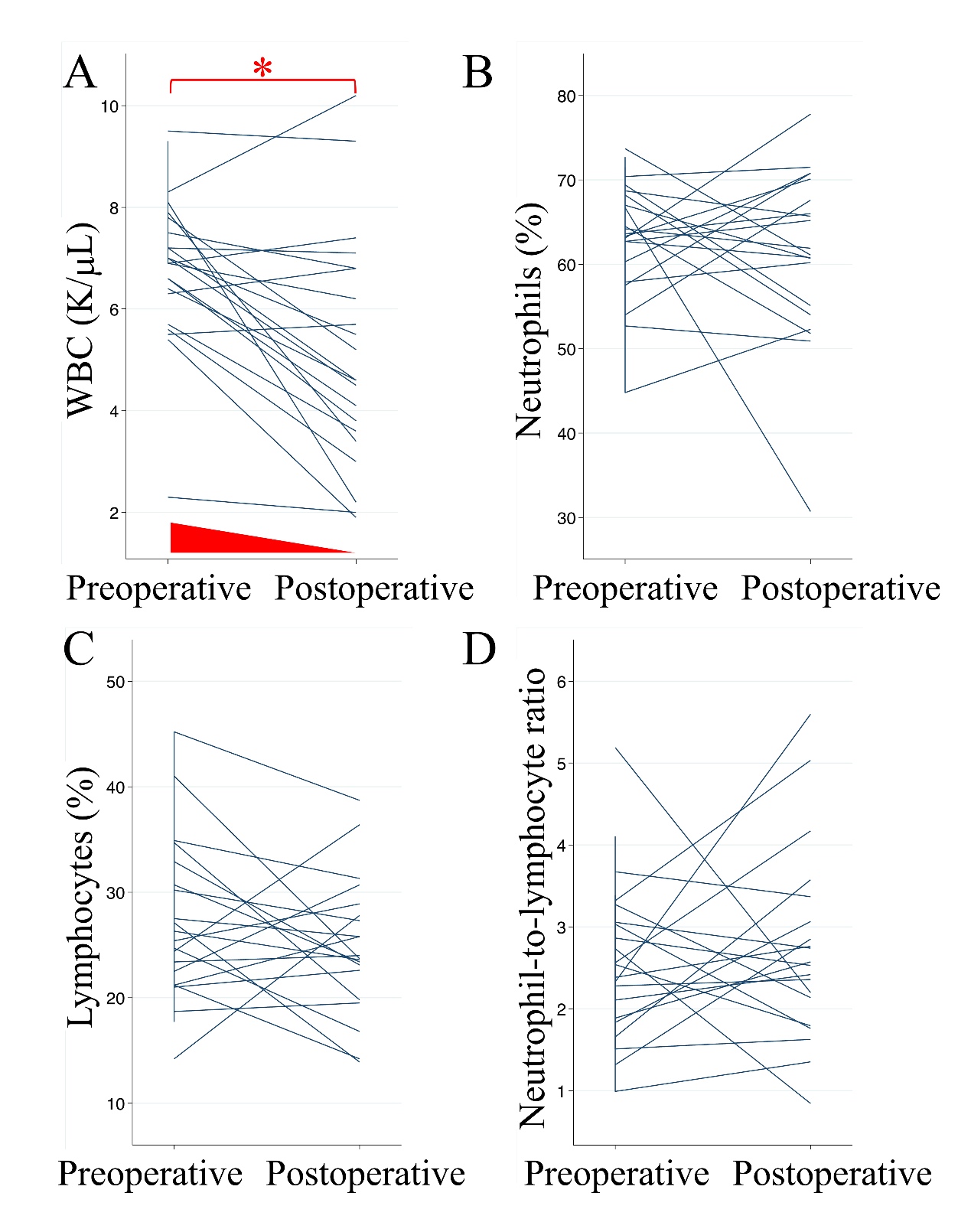


**Supplementary Figure 3.** Comparison between circulating immune cells in preoperative and postoperative data from the breast cancer testing group. (A) Total white blood cell count (*P* = 0.002), (B) neutrophil count (*P* = 0.416), (C) lymphocyte count (*P* = 0.416), and (D) Neutrophil-to-lymphocyte ratio (*P* = 0.416). The nonparametric Wilcoxon matched-pairs signed-ranks one-sided test was used for statistical analysis (**P* < 0.05).


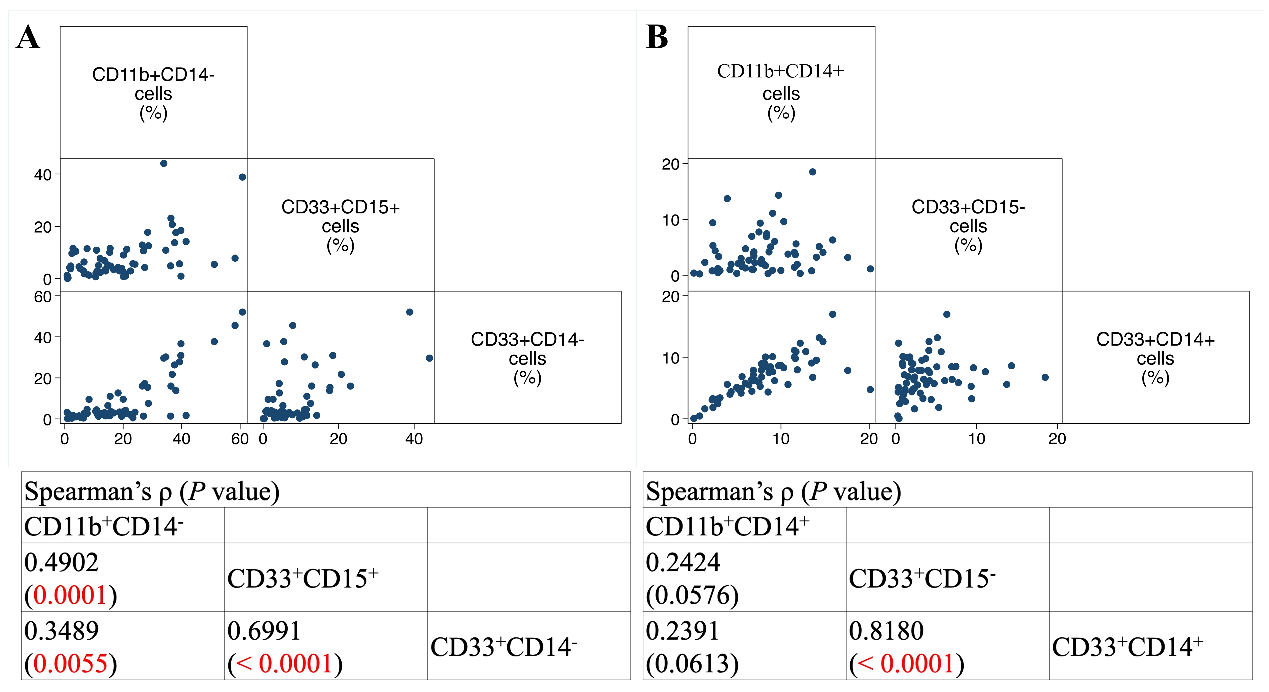


**Supplementary Figure 4.** Scatter plots depicting linear correlations between circulating immune cell subtypes in all participants. (A) PMN-MDSC (CD11b^+^CD14^−^, CD33^+^CD15^+^, and CD33^+^CD14^−^). (B) M-MDSC (CD11b^+^CD14^+^, CD33^+^CD15^−^, and CD33^+^CD14^+^). The upper numeral in each cell is correlation coefficient and the lower one is *P* value. Abbreviations: PMN-MDSC, polymorphonuclear myeloid-derived suppressive cell; M-MDSC, monocytic myeloid-derived suppressive cell.


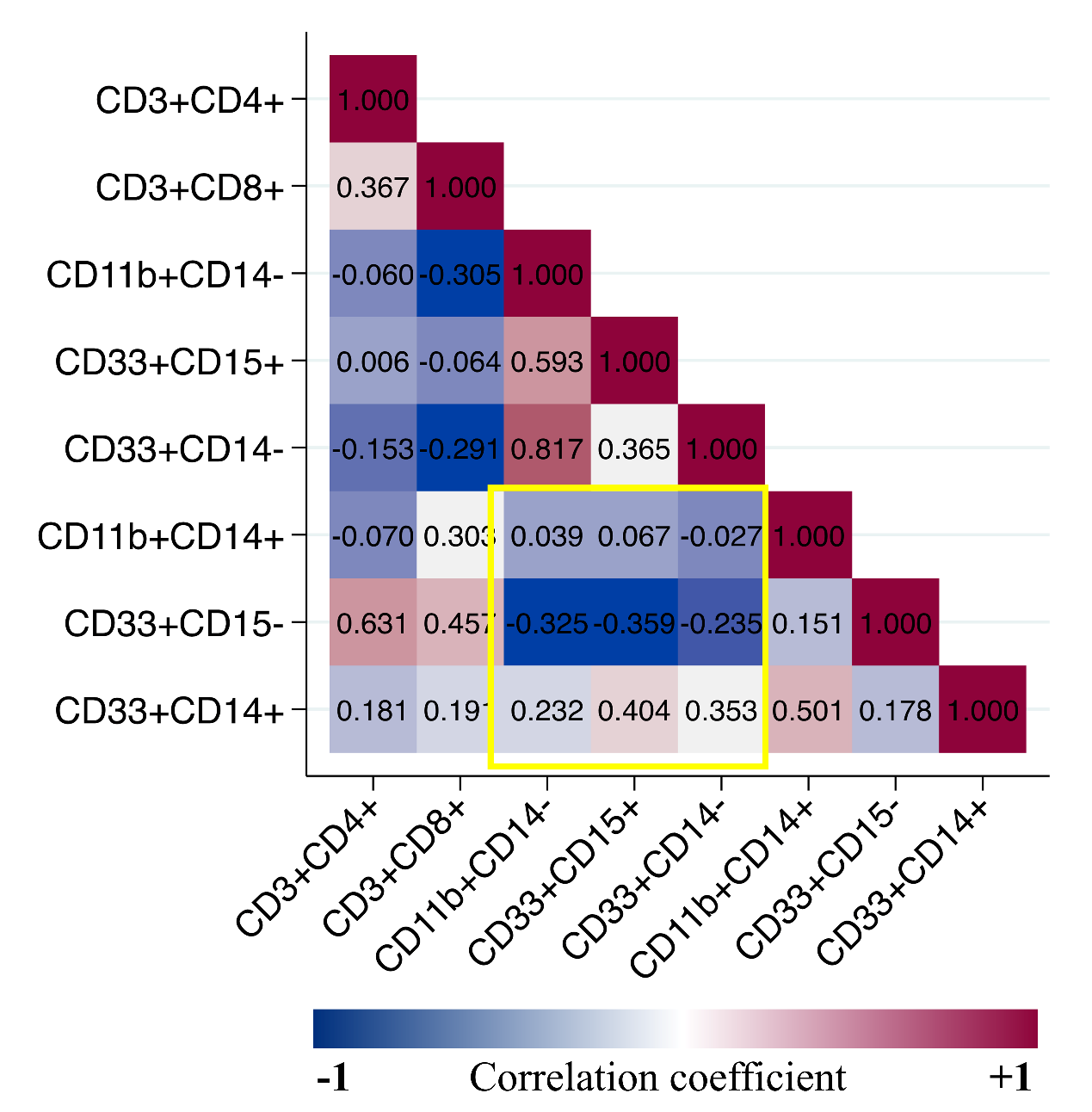


**Supplementary Figure 5.** Correlation matrix illustrating relationships between circulating immune cell subtypes. The numeral in each cell is correlation coefficient. Yellow box indicates correlations between PMN-MDSC (CD11b^+^CD14^−^, CD33^+^CD15^+^, and CD33^+^CD14^−^) and M-MDSC (CD11b^+^CD14^+^, CD33^+^CD15^−^, and CD33^+^CD14^+^).


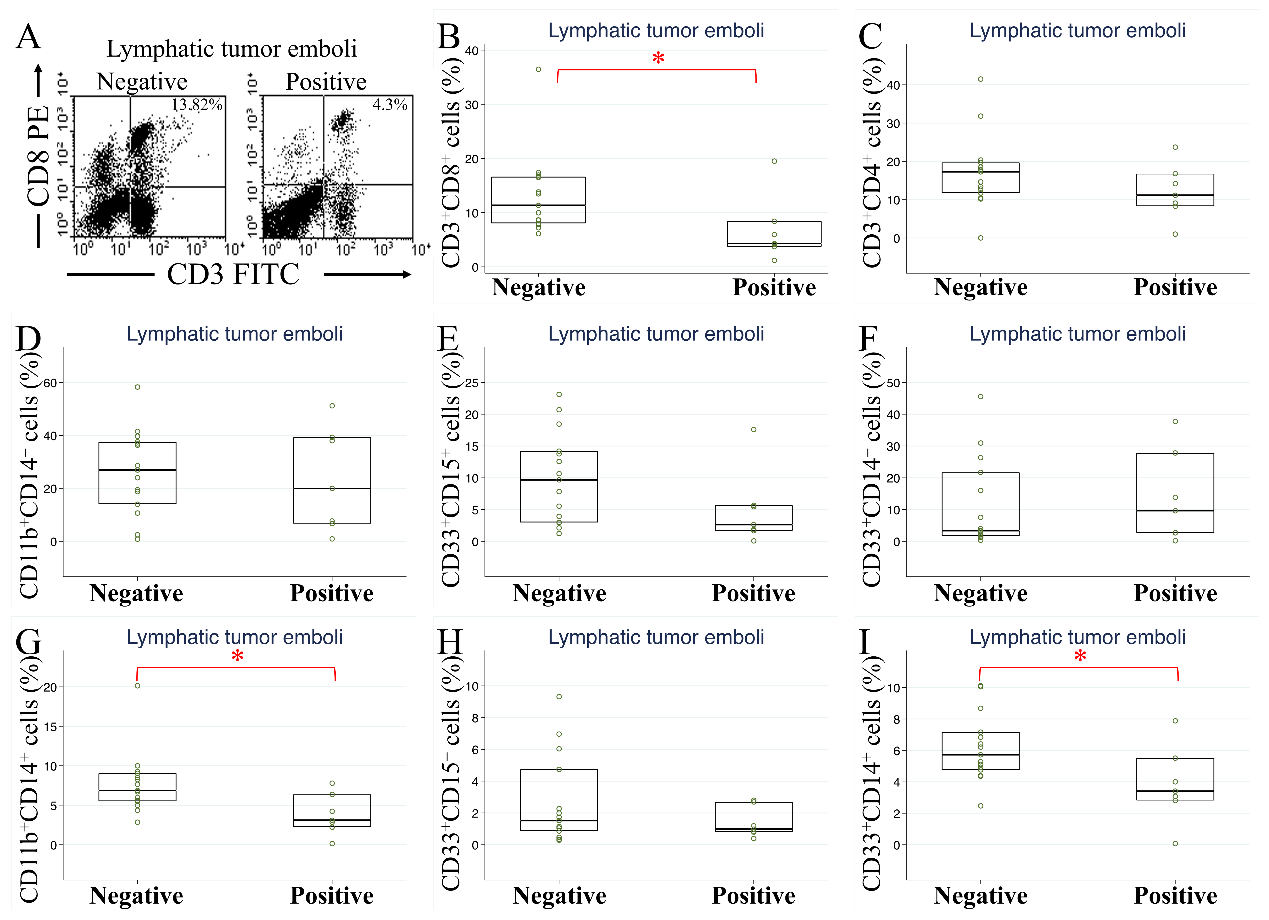


**Supplementary Figure 6.** Correlation of circulating immune cells with tumor stage lymphatic tumor emboli in the breast cancer testing group. (A) Example of double staining with CD3 and CD8 in flow cytometry. (B) CD3^+^CD8^+^ (*P* = 0.015), (C) CD3^+^CD4^+^ (*P* = 0.148), (D) CD11b^+^CD14^−^ (*P* = 0.805), (E) CD33^+^CD15^+^ (*P* = 0.072), (F) CD33^+^CD14^−^ (*P* = 0.751), (G) CD11b^+^CD14^+^ (*P* = 0.014), (H) CD33^+^CD15^−^ (*P* = 0.459), and (I) CD33^+^CD14^+^ (*P* = 0.038) cell populations. The nonparametric Mann–Whitney test was used for statistical analysis (**P* < 0.05).


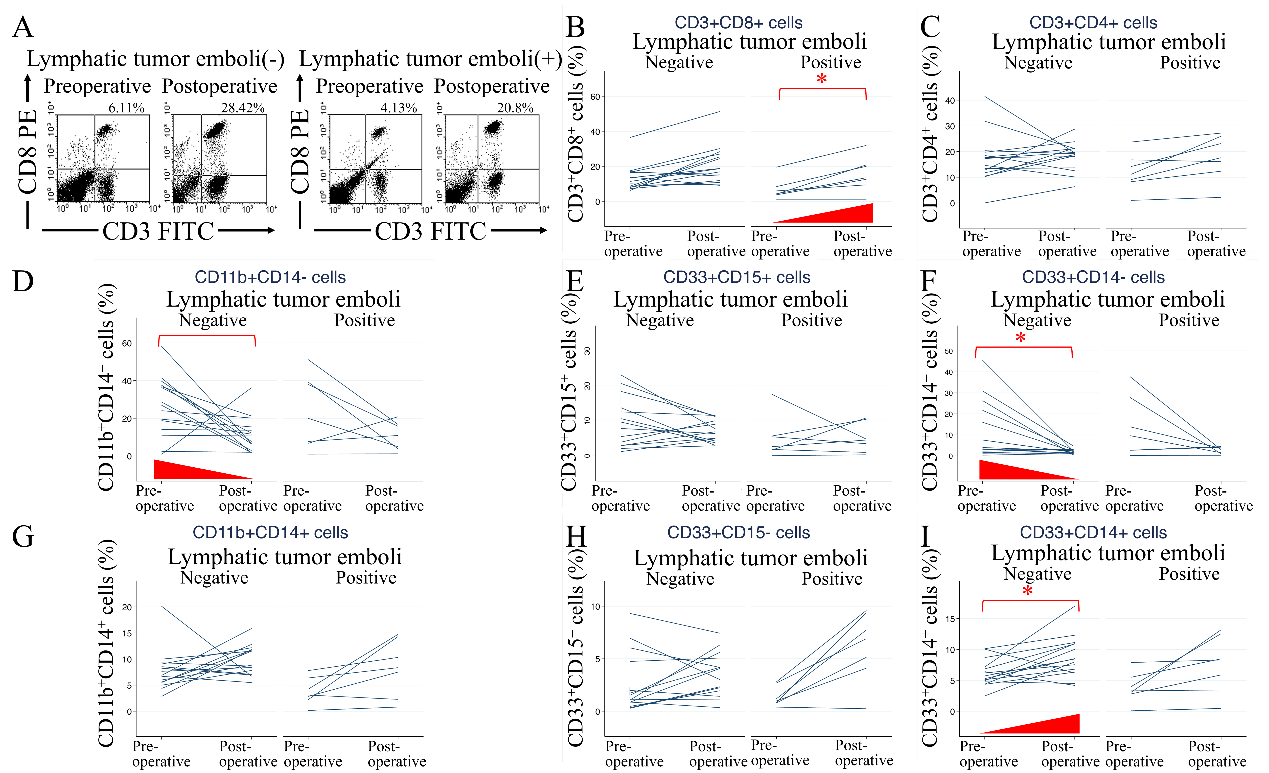


**Supplementary Figure 7.** Preoperative and postoperative circulating immune cells in patients without and with lymphatic tumor emboli (LTE). (A) Example of double staining with CD3 and CD8 cells in flow cytometry. (B) CD3^+^CD8^+^, (C) CD3^+^CD4^+^, (D) CD11b^+^CD14^−^, (E) CD33^+^CD15^+^, (F) CD33^+^CD14^−^, (G) CD11b^+^CD14^+^, (H) CD33^+^CD15^−^, and (I) CD33^+^CD14^+^ cell populations. The nonparametric Wilcoxon matched-pairs signed-ranks one-sided test was used for statistical analysis (**P* < 0.05).


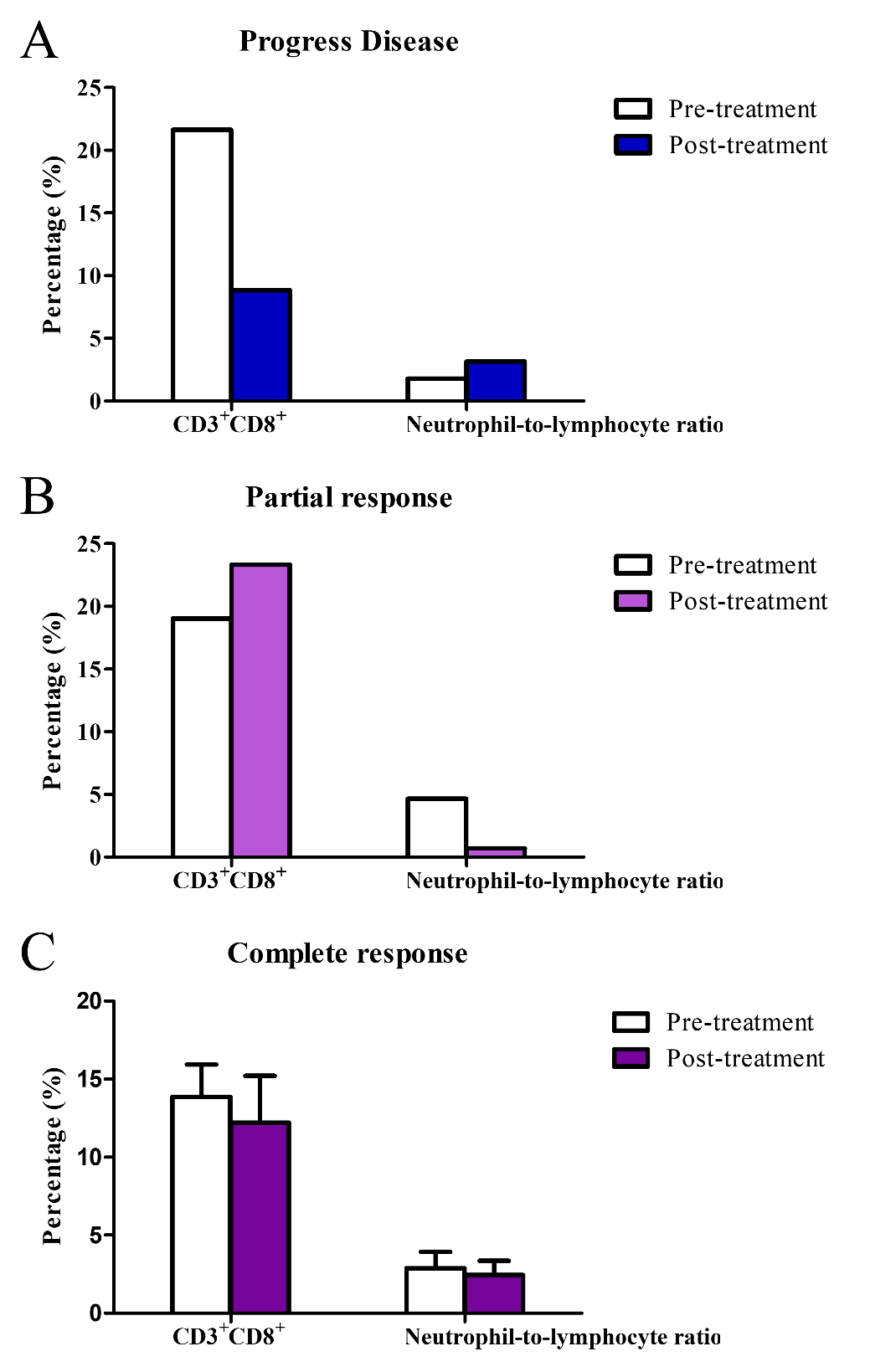


**Supplementary Figure 8.** CD3^+^CD8^+^ cell percentage and neutrophil-to-lymphocyte ratio in patients with breast cancer in the validation group (n = 5). Comparison between pretreatment and posttreatment levels. (A) Patient with progressive disease after standard treatment. (B) Patient with partial response and residual bone metastasis after standard treatment. (C) Three patients with complete response after standard treatment.

| **Supplementary Table 1** Proportion (%) of circulating immune cells in the healthy control and breast cancer testing group, shown as median (range). | | | |
| --- | --- | --- | --- |
|  | Healthy control  (n = 19) | Breast cancer patients  (Testing group, n = 22) | *P* |
| CD3^+^CD4^+^ | 24.52 (9.44–35.14) | 14.41 (0–41.49) | 0.007 |
| CD3^+^CD8^+^ | 15.84 (5.42–28.74) | 8.65 (1.19–36.47) | 0.038 |
| CD11b^+^CD14^−^ | 20.06 (2.07–60.79) | 25.48 (0.83–58.26) | 0.448 |
| CD33^+^CD15^+^ | 4.28 (0.71–44.05) | 5.60 (0.07–23.12) | 0.610 |
| CD33^+^CD14^−^ | 9.58 (0.52–52.10) | 3.68 (0.18–45.55) | 0.676 |
| CD11b^+^CD14^+^ | 7.79 (1.38–17.56) | 6.18 (0.14–20.13) | 0.214 |
| CD33^+^CD15^−^ | 3.44 (0.8–18.48) | 1.17 (0.28–9.32) | 0.002 |
| CD33^+^CD14^+^ | 6.75 (1.63–9.80) | 5.20 (0.07–10.11) | 0.147 |
